# A tale of two nematodes: Climate mediates mustelid infection by nematodes across the geographical range

**DOI:** 10.1016/j.ijppaw.2022.02.005

**Published:** 2022-02-09

**Authors:** Andrzej Zalewski, Marta Kołodziej-Sobocińska, Kamil A. Bartoń

**Affiliations:** a Mammal Research Institute, Polish Academy of Sciences, Stoczek 1, 17-230, Białowieża, Poland; b Institute of Nature Conservation, Polish Academy of Sciences, Al. A. Mickiewicza 33, 31-120, Kraków, Poland

**Keywords:** Host-parasite, Parasite prevalence, Geographic variation, Climate mediate interaction, *Aonchotheca putorii*, *Molineus patens*

## Abstract

Parasites have the potential to negatively affect host populations, if infection intensity is high. For parasites in which part of life cycle takes place outside the host, host infection intensity is likely affected by climate condition. Therefore, the parasite's impact on the host populations could be related to climatic conditions and may be altered with climate change. The aim of our study was to analyse the prevalence and infection intensity of two nematodes (*Aonchotheca putorii* and *Molineus patens*) from the Northern Hemisphere in relation to variations in climatic conditions. We reviewed 54 published studies on the occurrence of these two nematode species in 7 mustelid hosts. For *A. putorii*, infection parameters were higher when the stomach was included in the analyses compared to *M. patens.* The seasonality of precipitation influenced the prevalence the most, and the mean temperature of the warmest quarter had the strongest influence on infection intensity. The predicted prevalence of *M. patens* increased with increasing seasonal variation in precipitation, while the prevalence of *A. putorii* decreased. The predicted infection intensity of *M. patens* decreased with increasing precipitation seasonality, whereas the intensity of *A. putorii* infection did not change much. *A. putorii* infection intensity significantly decreased with increasing mean temperature of the warmest quarter, while the infection intensity of *M. patens* was not significantly related to this variable. Prevalence and infection intensity varied over the geographic range for both parasites, broadly with higher levels in northern latitudes for *A. putorii* and in southern latitudes for *M. patens*. Our study highlights the differences between these two nematode species and shows that the severity of host infection by these parasites is complex and mediated by climatic conditions. The results suggest that current climate change may potentially modify susceptibility and exposure to parasitic infections in mustelids.

## Highlights

• Analyses of the infection intensity of two nematodes on seven mustelid species on a large geographical scale.
• Climate modulates the infection parameters of the studied nematodes.
• Climatic conditions have contrasting effects on the two nematode species.
• The different effect of climate on parasite infection intensity is related to differences in their life cycles.
• Climate change may modify parasite infections and parasite impacts on wild carnivores.

## Graphical abstract

**Figure:**
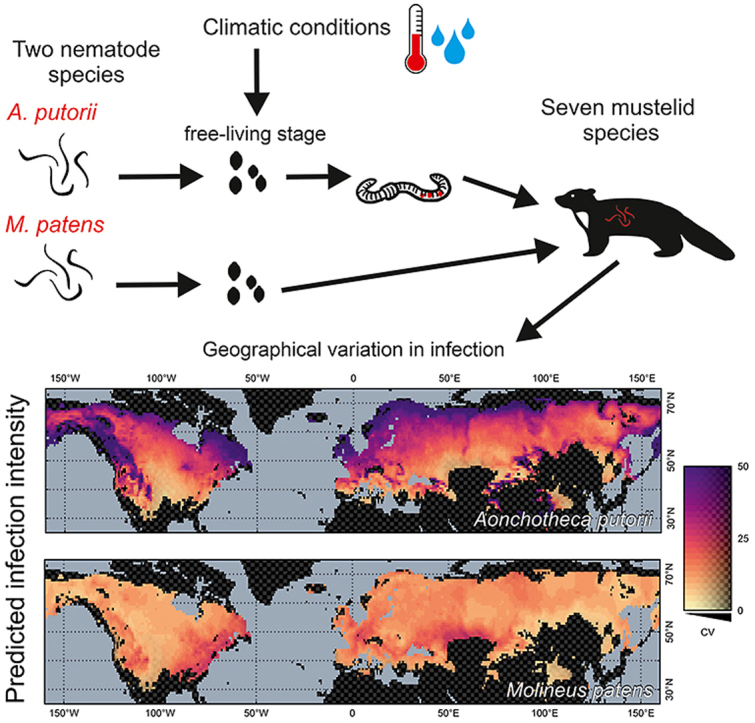

## 1 Introduction

Parasites have the potential to regulate host population abundance; however, their influence may be related to the species of parasite as well as infection intensity (Tompkins et al., 2002). The influence of parasites on populations may take various forms, for example, by reducing host fitness, e.g. through lowering host body condition, but also by changing host behaviour or vulnerability to predation (Beldomenico et al., 2008; Kołodziej-Sobocińska, 2019; Kołodziej-Sobocińska et al., 2018; Thomas et al., 1998). Some parasites are specific to a particular host species and the circulation of these parasites are therefore related to the dynamics of a single host population (e.g. Popiołek et al., 2011). However, most parasite species can infect multiple host species within the same family (e.g. Sulimov, 1968; Torres et al., 2008). Parasite infection intensity in these hosts is related to infection probability, which in turn is related to the biology and ecology of the host (e.g. diet composition, habitat selection) and the life cycle of the parasite. For parasites in which part of the life cycle takes place outside the host, their survival and subsequent host infection probability are likely to be affected by weather and climatic conditions (Charlier et al., 2016; Hernandez et al., 2013; Ogden and Lindsay, 2016; Rose et al., 2014). Infection parameters (prevalence and infection intensity) may, therefore, vary spatially (Charlier et al., 2016; Poulin and Dick, 2007).

Parasitic nematodes infect a broad range of hosts, including plants, wild and domestic animals, and humans (Blaxter et al., 1998) and their role in affecting host populations is becoming increasingly concerning (Arneberg et al., 1997; Coulson et al., 2018). Terrestrial nematodes usually have a simple life cycle, in which adults produce thick-shelled eggs, the infected host defecates them into the environment and another host of the same species acquires the eggs or larvae with food and/or water. With such a simple life cycle, the abundance of released eggs in the environment is partially related to the intensity of host infection. The survival and persistence of eggs or larvae are both also related to environmental conditions, mainly ambient temperature and humidity, and is often higher in warm and humid environments (Froeschke et al., 2010; Rodel and Starkloff, 2014; Schai-Braun et al., 2019; Stromberg, 1997). In the Nearctic and Palearctic, seasonal variations in these two environmental parameters may also affect the mortality of free-living parasite stages, e.g. larval survival of rotostrongylid nematodes decreases with freezing duration (Kafle et al., 2018). In contrast, infection intensity by nematodes with complex life cycle, in which larvae develop in intermediate hosts, may be less dependent on environmental conditions. Therefore, in northern latitudes, the prevalence and infection intensity of nematodes with a simple life cycle should be lower compared to more southern latitudes where more favourable climatic conditions for parasite free-living stages enhance host exposure to infections. However, generalist parasite infection intensity is often studied over relatively small parts of their range as studies of one host species often cover only part of the specific parasite distribution. As a result, patterns of host infection levels throughout large parasite geographic ranges are poorly understood (Poulin and Dick, 2007). For a comprehensive analysis, the inclusion of various host species from a large part of the parasite range is required.

Two nematode species, *Aonchotheca putorii* (*Capillaria putorii* of previous authors; Capillariidae) and *Molineus patens* (Molineidae), occur on many continents and infect many carnivore species, mainly mustelids (Anisimova, 2004; Foreyt and Lagerquist, 1993; Nugaraite et al., 2019; Odnokurtsev and Sedalischev, 2011; Torres et al., 2004, 2008). In many mustelids, the prevalence of both nematodes is often high, reaching up to 97% (e.g. Kretschmar, 2016; Liberge, 2004; Schoo et al., 1994); these two parasites often dominate the entire parasite community (e.g. Ivonin and Ivonina, 2019; Kontrimavichus, 1969; Kretschmar, 2016; Martínez-Rondán et al., 2017). Our previous analyses have shown that these nematodes heavily infect the introduced American mink (*Neovison vison*) in Europe, and the severity of infection results in reduced body condition of the mink, probably affecting its population sizes (Brzeziński et al., 2019; Kołodziej-Sobocińska et al., 2018, 2021). This suggests that these nematodes (especially *A. putorii*) may have a negative impact on mustelids in general. Although the data on these nematodes in various host species have been constantly accumulating in the literature, comparisons of *A. putorii* and *M. patens* infections across the distribution of these parasites are lacking. This gap stems from scarce knowledge of the biology and ecology of both nematodes, including a lack of analysis of the climatic conditions influencing egg and larval survival in the environment. The life cycle of *M. patens* is direct (Gupta, 1961). In contrast, earthworms serve as reservoir hosts in the life cycle of *A. putorii* and even the life cycle of *Capillaria eriancei* (which is a synonym of *A. putorii*; Butterworth and Beverley-Burton, 1980) requires earthworms for the development of infection in the definitive host (Romashov, 1980). This indicates that *A. putori* displays a complex life cycle (potentially less dependent on environmental conditions) as opposed to *M. patens* which displays a simple life cycle (more dependent on climate).

The aim of our study was to compare infection intensity of two nematode species (*A. putorii* and *M. patens*) in hosts from the Northern Hemisphere, in relation to climatic conditions. To do this, we undertook a literature review of the occurrence of these two nematode species in mustelids. From a variety of mustelids infected by these parasites, we selected seven species: American mink, European mink (*Mustela lutreola*), European polecat (*Mustela putorius*), sable (*Martes zibellina*), pine marten (*Martes martes*), stone marten (*Martes foina*) and American marten (*Martes americana*), which are similar in size (weight on average approx. 1 kg). We selected these species to avoid the confounding effect of body size on prevalence. Thus, we chose mustelids inhabiting large geographic ranges all together covering a large part of the ranges of both parasites. We expected that, in colder climates, prevalence and infection intensity would be lower than in warmer climates, while in environments with higher humidity infection parameters would be higher than in low humidity environments, in both parasite species. Both nematodes inhabit all parts of the alimentary tract; however, *A. putorii* mainly resides in the stomach, whereas *M. patens* resides in the intestine (Martínez-Rondán et al., 2017; Nugaraite et al., 2014; Torres et al., 2004). Therefore, the material (specific parts of the digestive tract) used in the parasitological studies may confound our analysis. For this reason, we also evaluated the influence of this factor on the infection parameters in mustelid host species.

## 2 Methods

We searched multiple bibliographic databases for publications on the parasites *Aonchotheca putorii* (*Capillaria putorii* of previous authors and syn. *C. mustelorum*) and *Molineus patens* in the seven selected mustelid species. The databases included Google Scholar, SCOPUS, Web of Science, JSTOR, and ProQuest. Articles were searched for the terms ‘putorii’, ‘Aonchotheca’ ‘Capillaria’, ‘mustelorum’, ‘Molineus patens’ or ‘patens’ and all query results were verified manually by selecting only the papers concerning mustelids from the genera *Martes*, *Mustela* and *Neovison*. We did not include weasels or stoats (*Mustela erminea*), as these species are much smaller, nor the Japanese marten (*Martes melamus*) because only two studies described parasite infections in this species. Next, we reviewed the references in all papers found (especially in Russian) to find other papers/books that were not included in the databases and we searched for them in the Mammal Research Institute of the Polish Academy of Sciences library or ordered them from other libraries (20% of all used papers).

We excluded two papers because they were based on analysis of fewer than six host individuals, and papers analysing parasite infection based on scats. We used two parameters of nematode infection: (1) parasite prevalence (number of infected hosts/total number of hosts); (2) mean infection intensity (mean number of parasites per host individual, excluding uninfected hosts; Bush et al., 1997).

The comparison of infection parameters among studies may be confounded by various factors. First, nematodes occur in the stomach; however, some papers only use the term intestine (e.g. Zhigileva and Uslamina, 2016) or did not include information regarding the use of stomachs in the description of the methods. Furthermore, in some studies, diet analyses were performed simultaneously with parasite studies and the stomach was probably not included in the parasitological investigations (e.g. Anisimova, 2002; Jennings et al., 1982). To account for this, we added a binary variable to the model, specifying whether the stomach was used in the study or no information on the gastrointestinal parts used was provided. Second, as the number of host individuals studied (sample size) could affect the accuracy of results on prevalence and infection intensity, we included sample size (scaled to sum to the total number of observations) as prior weights in the model.

Next, we analysed the effects of climatic variables, host species and sampling material (parts of the digestive tract used in the analysis) on infection prevalence and intensity of the two nematode species. Global bioclimatic variables, derived from monthly temperature and precipitation values according to the ANUCLIM scheme (Abatzoglou et al., 2018), were obtained from WorldClim (version 2.1; Fick and Hijmans, 2017) as an average for 1970–2000 at 30 s spatial resolution. Out of the 19 standard bioclimatic variables, we selected eight reflecting temperature and precipitation (average over the whole year, coldest and warmest quarters, and their seasonality) as they were likely to affect the infection parameters (Schai-Braun et al., 2019; Stromberg, 1997). Seasonality reflects the variability of a climatic variable within a year and is taken to be the standard deviation of monthly averages multiplied by 100 for temperature and the coefficient of variation of monthly values for precipitation. To account for the uncertainty in the location of each study, the bioclimatic variables used were averaged over a circular area of 50 km^2^ around the assigned study location, as some studies covered a large area. In the initial analyses, we used buffers from 25 to 100 km^2^, but we found that varying the buffer size made little difference in the resulting models. Precipitation-related variables were square-root transformed to linearise the relationship with the response.

For each of the two infection parameters, we first fitted a ‘global’ model including the effects of all considered explanatory variables, specific for both parasite species. These models included the stomach presence (a binary variable), host species (a categorical variable with seven levels) and eight bioclimatic variables, and were parameterised to include separate slope parameters for each nematode species and explanatory variable, meaning that the nematode species effect was retained in all sub-models and all other variables were included as their interaction with parasite species. In the preliminary analysis, we also assessed interactions between the explanatory variables (other than parasite species), but found that these did little to improve the models and they were therefore removed. To model prevalence, we used beta regression with a complementary log-log link, chosen based on the comparison of models with alternative link functions, and Generalized Linear model (GLM) with a Gamma error distribution and logarithmic link for modelling the mean infection intensity. To identify outliers, we used Cook's distance, and iteratively downweighted by 50% the observations that were disproportionally influential in the global models (Cook's distance above the 95% quantile and greater than 0.3; values were chosen to conservatively identify the outlying observations). This reduced the effective number of observations to 129 out of 131, and 97 out of 102, respectively for the prevalence and intensity models.

We then performed all-subsets regression by generating models containing all combinations of the explanatory variables. We limited to models with up to five explanatory variables (and their interaction with parasite species) because of the relatively small number of observations. Multicollinearity was assessed with VIF (Fox and Weisberg, 2018); on this basis we further excluded models containing correlated explanatory variables (i.e. models with (G)VIF ≥5). The resulting sets of models were ranked with AIC_c_. Since, for both dependent variables, there was no clearly ‘best’ model, we averaged models with ΔAIC_c_≤ 4 (Burnham and Anderson, 2002). We present the averaged model coefficients and effect plots for each explanatory variable included in the final model to show differences between the nematode species. We report approximate significance values based on the model-averaged variance and confidence intervals (Burnham and Anderson, 2002). Based on the averaged models, we also made predictions of both infection rates in the Northern Hemisphere, limited to the area covering the range of the bioclimatic variables used to fit the model. Model predictions were averaged on the response scale (i.e. untransformed). In calculating the predictions, we assumed that the stomach was included in the studies.

All analyses were performed in R version 4.0.0 (R Core Team, 2013), using packages ‘betareg’ (Grun et al., 2012) for beta-regression, ‘MuMIn’ (Bartoń, 2020) for model averaging, ‘effects’ (Fox and Hong, 2009) for the effect plots (adapted for averaged models) and ‘MATA’ (Turek, 2021) for model-averaged tail area confidence intervals. The figures were created using R's base graphics.

## 3 Results

**Fig. 1:**
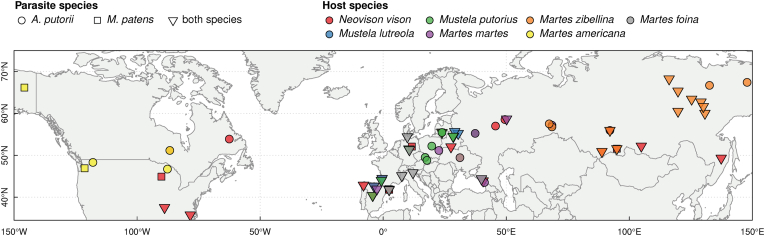
Geographical location of study sites for the nematode parasites *Aonchotheca putorii* and *Molineus patens*, within the Palaearctic and Nearctic in seven mustelid host species. Details of the samples are given in Table S1.

We collected data from 54 papers describing infection parameters by one or both nematode species (*Aonchotheca putorii* and *Molineus patens*) from 58 sites, in at least one of the seven analysed mustelid host species (Fig. 1; see details in Supplementary materials, Table S1). A simple test for correlations between both infection parameters showed that in all hosts, the intensity of infection was positively related to the prevalence of *A. putorii* (r_p_ = 0.668, p ≪ 0.0001) but not to *M. patens* (r_p_ = 0.22, p = 0.2), which may be because of the smaller range of both variables in the latter species (Fig. S1).

**Fig. 2:**
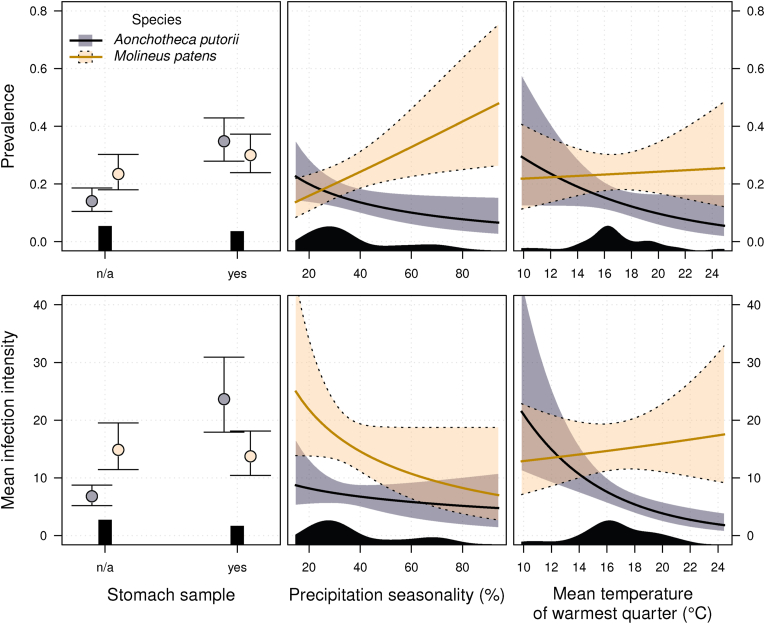
Model results for *Aonchotheca putorii* and *Molineus patens* infection parameters in seven mustelid species. Panels show model-averaged predictions of prevalence and infection intensity depending on the part of the alimentary tract analysed and climatic conditions (seasonality in precipitation and mean temperature of the warmest quarter). Points and lines show the mean, whiskers or shading denote 95% confidence intervals. Remaining explanatory variables were kept at their mean values or most common factor levels. The distribution of the data is shown at the bottom axes as a histogram or density plots.

For *A. putorii*, average predicted prevalence and infection intensity were 2.5 and 3.5 times higher, respectively, when the stomachs were included in the analysis compared to those in which the analysed parts of the alimentary tract were not specified (Fig. 2, Tables 1 and S2). In contrast, no significant differences were found in *M. patens* between these two groups (Fig. 2, Table 1). In addition, the prevalence and infection intensity were higher for *A. putorii* than for *M. patens* when stomachs were analysed, while the opposite was true when the analysed parts of the alimentary tract were not specified, as evidenced by a significant interaction between species and analysed stomach (1.32 ± 0.24, p ≪ 0.0001). The predicted average prevalence of *A. putorii* and *M. patens* was 0.35 and 0.30, and the predicted averaged infection intensity was 23.6 and 13.7 parasite individuals per host respectively, when the stomach was included in the analyses. For both parasite species, there were no significant differences in the infection intensity among host mustelid species.

**Table 1:** The effect of the part of the alimentary tract analysed and climatic variables on the prevalence and infection intensity of mustelids by two nematode species: *Aonchotheca putorii* (Ap) and *Molineus patens* (Mp) from averaged model estimates. Coldest Q and warmest Q – coldest and warmest quarters of the year, respectively. *p < 0.05, **p < 0.01, and ***p < 0.001.

Parameter description	Estimate ± SE	z	P	
**Averaged model coefficients for prevalence (ΔAIC**_**c**_ ≤ **4)**				
Intercept	1.806 ± 1.14	1.59	0.112	
Between-species effect	−4.502 ± 1.61	2.81	0.005	**
Stomach effect in Ap	1.040 ± 0.19	5.48	≪ 0.001	***
Stomach effect in Mp	0.289 ± 0.20	1.47	0.14	
Precipitation seasonality effect in Ap	−0.226 ± 0.11	2.09	0.036	*
Precipitation seasonality effect in Mp	0.254 ± 0.10	2.60	0.009	**
Temperature of coldest Q effect in Ap	0.011 ± 0.02	0.68	0.497	
Temperature of coldest Q effect in Mp	0.015 ± 0.02	0.76	0.447	
Temperature of warmest Q effect in Ap	−0.121 ± 0.07	1.82	0.069	.
Temperature of warmest Q effect in Mp	0.012 ± 0.05	0.25	0.804	
Temperature seasonality effect in Ap	0 ± 0	0.68	0.497	
Temperature seasonality effect in Mp	0 ± 0	0.73	0.463	
Average temperature effect in Ap	0.015 ± 0.03	0.52	0.603	
Average temperature effect in Mp	0.020 ± 0.04	0.55	0.584	
**Averaged model coefficients for mean infection intensity (ΔAIC**_**c**_ ≤ **4)**				
Intercept	5.680 ± 1.09	5.22	≪ 0.001	***
Between-species effect	−2.090 ± 1.53	1.37	0.17	
Stomach effect in Ap	1.240 ± 0.16	7.87	≪ 0.001	***
Stomach effect in Mp	−0.079 ± 0.18	0.43	0.67	
Precipitation seasonality effect in Ap	−0.103 ± 0.13	0.79	0.43	
Precipitation seasonality effect in Mp	−0.217 ± 0.14	1.54	0.12	
Average temperature effect in Ap	0.013 ± 0.02	0.56	0.57	
Average temperature effect in Mp	−0.009 ± 0.02	0.48	0.63	
Temperature of warmest Q effect in Ap	−0.169 ± 0.05	3.63	≪ 0.001	***
Temperature of warmest Q effect in Mp	0.021 ± 0.04	0.55	0.58	

Of the eight bioclimatic variables considered, seasonality of precipitation (larger values represent greater variability of precipitation over the course of the year) and mean temperature of the warmest quarter had significant effects on infection parameters (Table 1). Prevalence was most influenced by precipitation seasonality, positively in *M. patens* and negatively in *A. putorii* (Fig. 2, Table 1). The predicted prevalence of *M. patens* increased from 0.14 (95% CI = 0.08–0.22; hereafter, the values in parentheses refer to the 95% CI) to 0.48 (0.26–0.75) with increasing variation of seasonal precipitation while prevalence of *A. putorii* decreased from 0.23 (0.14–0.35) to 0.07 (0.03–0.15; Fig. 2). The model did not show a relationship of prevalence with mean temperature of the warmest quarter. Conversely, predicted infection intensity was related mostly to the temperature of the warmest quarter, but only in *A. putorii* (Table 1). With the increase of mean temperature of the warmest quarter from 9.8 °C to 24.9 °C, the infection intensity of *A. putorii* decreased from 21.4 (11.34–43.79) to 1.8 (0.83–3.81), and infection intensity in *M. patens* increased from 12.9 (7.12–22.84) to 17.5 (9.18–32.85; Fig. 2). The predicted infection intensity of *M. patens* decreased with increasing variation of seasonal precipitation from 24.9 (13.87–44.14) to 7.0 (2.7–18.74).

**Fig. 3:**
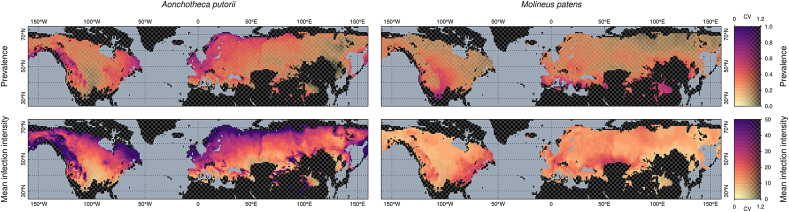
Prediction of prevalence and infection intensity of *Aonchotheca putorii* and *Molineus patens* in the Northern Hemisphere based on averaged model estimates, using the WorldClim climate data set. Areas outside of the range of climatic variables used for model fitting are filled with checkerboard pattern. Transparency of the prediction colour reflects the coefficient of variation (CV) of predicted values (i.e. more uncertain prediction has more pattern tint). Predictions assumed the stomach was included in the analyses. (For interpretation of the references to colour in this figure legend, the reader is referred to the Web version of this article.)

Predicted values of prevalence and infection intensity varied over the geographic range of both parasites (Fig. 3). For *A. putorii*, prevalence and infection intensity were higher in regions with lower seasonal variation of precipitation and lower mean temperature of the warmest quarter, e.g. Scandinavia and central Europe and lower in eastern Siberia and central parts of North America. For *M. patens*, the geographical variation in both infection parameters were lower and less pronounced. The prevalence increased in regions of higher seasonal variation of precipitation and lower mean temperature of the warmest quarter, e.g. southern Europe, but the infection intensity increased in regions of lower seasonal variation of precipitation, e.g. central Europe or the east coast of North America (Fig. 3).

## 4 Discussion

The results of our review show little variations in *A. putorii* and *M. patens* infection rates among the seven mustelid species. Climatic conditions had a contrasting impact on these nematode species: seasonal variation in precipitation strongly affected the infection prevalence of *M. patens* and partly of *A. putorii*, whereas the temperature of the warmest quarter affected the infection intensity of *A. putorii* only. We also showed that *A. putorii* infection intensity was higher than that of *M. patens*, which suggests that *A. putorii* may have a more severe impact on the host or indicate a less detrimental relationship between this parasite and the host organism.

To cover much of the range of both parasites, our review included seven mustelid species inhabiting areas spanning high climatic variation, and occupying various habitats: polecat, European and American mink inhabit various riparian habitats; American marten and sable occupy coniferous forest in boreal regions; pine marten is found in deciduous and mixed forests; and stone marten often live in anthropogenic areas (cities and villages) in eastern and central Europe, as well as natural, often fragmented, forests and agricultural habitats in south-eastern Europe and the Mediterranean. These differences in host ecology may have increased the variation in the results of our study. Nevertheless, we observed relatively little variation in *A. putorii* and *M. patens* infection intensity among host species. We demonstrated, however, that the parts of the digestive tract used in analyses could bias the estimated infection prevalence and intensity. The methods used in parasitological studies strongly affected the results for *A. putorii*, but not for *M. patens*, which is concordant with the biology of these nematodes (e.g. Kretschmar, 2016). Studies not including information on whether stomachs were used in the parasitological analyses underestimated the parameters of *A. putorii* infection, as this species inhabits mainly the stomach. In *M. patens*, inhabiting mainly the intestines, such a relation was not observed. Including the material type used in analyses partially allowed us to compare the infection parameters between sites and host species, but this bias should be avoided in future studies by including the stomach in parasitological analyses.

This study revealed complex relationships among climatic conditions and parasite infection. While the seasonality of precipitation negatively correlated with overall infection intensity in both parasite species, the prevalence of *M. patens* showed a positive relation with the seasonality of precipitation. In contrast, the prevalence and infection intensity of *A. putorii* were negatively related to temperature in the warmest quarter of the year, whereas *M. patens* infection parameters were not affected.

This shows that in the Northern Hemisphere climate affects infection in mustelids differently depending on the parasite species, such that infection parameters are higher in Northern latitudes for *A. putorii* but in southern latitudes for *M. patens*. Differences in how these parasites have adapted to environmental conditions may underlie differences in infection parameters between them. For *A. putorii*, infection parameters were higher in sites with a colder climate, but temperature did not affect the infection parameters of *M. patens*. The differences in the biology of the two parasites may explain these contrasting patterns. The mortality of infective stages living outside their hosts is often assumed to be higher at higher latitudes as a result of harsh winter conditions (Nunn et al., 2005), so we should expect that climatic conditions, which best describe the dynamics of free-living stages, have a linear impact on host infection. Our knowledge of the biology and ecology of these two parasite species is only fragmentary. However, the experimental study of the life cycle of *Capillaria eriancei* (which is a synonym of *A. putorii*; Butterworth and Beverley-Burton, 1980) revealed that earthworms (reservoir hosts) are necessary for the development of infection in the definitive host, the hedgehog (*Erinaceus europaeus*) (Romashov, 1980). This indicates that *A. putori* displays a complex life cycle. Otherwise, for *M. patens*, a simple life cycle with eggs being the source of infection directly from the contaminated environment has been demonstrated (Gupta, 1961, 1963). These features are important when assessing the impact of climatic conditions on the infectivity and survival of both parasites. Earthworms may survive even severe winters and higher seasonal variation in precipitation underground, meaning greater survival of *A. putorii* larvae, in contrast to *M. patens* larvae which are exposed directly to climatic conditions when deposited on grass, soil and/or water. This is consistent with our results showing that the infection parameters of *A. putorii* infection are on average higher in northern latitudes than those of *M. patens* in the mustelid hosts. The diet of the mustelid species ranged from exclusive carnivores (mink and polecats) to omnivores with a large proportion of fruits in the diet (stone marten) (Bartoszewicz and Zalewski, 2003; Czernik et al., 2016; Zalewski, 2004; Zalewski et al., 2021; Zhou et al., 2011). Oligochaetes may be present in the diet of all mustelids, but this food item is often overlooked and rarely reported in studies of the predator diet. It is therefore difficult to assess the importance of the impact of this prey in the circulation of *A. putorii*.

In *A. putorii*, infection intensity strongly correlated with prevalence. This is contrary to other studies that showed a rather weak relationship between prevalence and infection intensity (Arneberg et al., 1997; Poulin, 2006). Poulin (2006) explained the lack of relationship between these parameters by the difference in the factors influencing them. Infection intensity is partly related to the density-dependent survival of the parasite within the host, whereas prevalence is related to the rate of parasite-host encounters, both of which depend mainly on processes outside the host (local environmental conditions). Our results may suggest that the survival rate of *A. putorii* in mustelids does not depend on parasite density in individual hosts because of e.g. low intraspecific competition. Increasing numbers of transmission events (probably related to egg/larvae survival) of *A. putorii* affected the increase in proportion of hosts infected (prevalence) and the average infection intensity of one host to the same extent. The effect of precipitation seasonality on *M. patens* infection was the opposite: we observed a positive impact on infection prevalence and a negative effect on infection intensity, which may explain the lack of association between these two parameters. This finding is difficult to explain based on current knowledge, but highlights the differences in the biology of these two parasites.

Both nematode species are common parasites of mustelids. Besides the seven analysed species, they infect other Mustelidae, as well as Canidae, Procyonidae, Ursidae and Gliridae (Borgsteede, 1984; Durette-Desset and Pesson, 1987; Jones et al., 1980; Kozlov, 1963, 1977; Rataj et al., 2013; Rausch et al., 1979; Sato et al., 1999; Shimalov and Shimalov, 2001; Thiess et al., 2001). In many mustelids, these two parasites dominate the entire parasite community affecting them (e.g. Ivonin and Ivonina, 2019; Kontrimavichus, 1969; Kretschmar, 2016; Martínez-Rondán et al., 2017). However, these nematodes are rarely studied, and our knowledge of the biology of *A. putorii* and *M. patens* and their impact on host species is still limited, despite their potentially important effects on mustelids and other carnivores. Most *A. putorii* individuals concentrate in the stomach, a relatively small part of the gastrointestinal tract, whereas *M. patens* is distributed along the intestines (Kołodziej-Sobocińska et al., 2021). Furthermore, in some cases, infection with *A. putorii* was very high (e.g. 2445 specimens per host; Kontrimavichus, 1969). This suggests that *A*. *putorii* may have a stronger negative or a less detrimental impact on its hosts than *M*. *patens* and previous studies have shown that the former outcome is more likely. Firstly, the body condition of American mink was lower in individuals highly infected by nematodes, which were mainly concentrated in the stomach (Kołodziej-Sobocińska et al., 2018). Second, *A. putorii* infection in domestic cats can have severe consequences: chronic gastritis, vomiting, diarrhoea and weight loss (Curtsinger et al., 1993; Nakanishi and Nakanishi, 2013). However, the data on the lack or negative impact of *A. putorii* on hosts are scarce, limiting our ability to determine the effects of this parasite on the host species.

Due to the heterogeneous influence of climatic conditions on the prevalence and infection intensity of these two parasite species in their mustelid hosts, the impact of infection varies across their geographic range, dependent on parasite species. Higher infection intensity of *A. putorii* in northern latitudes may reduce body condition and fitness of mustelids in these areas, and as a consequence, contribute to the reduction of host densities. This impact should be considered in the analyses of factors affecting population density in relation to climate conditions (e.g. Bartoń and Zalewski, 2007), as these may, at least partially, affect animal density through an increased pressure of parasites. Various models projected different scenarios of climate change but in general the rate of climate change is increasing toward the north (e.g., Swaminathan et al., 2022). Based on these scenarios, our results suggest that climate warming may reduce infection intensity in wild mammals of *A. putorii* but increase intensity of *M. patens*; however, these changes may vary in different areas due to changes in temperature and seasonal rainfall fluctuations.

## 5 Conclusions

Our study highlights the differences in the prevalence and infection intensity of mustelid species by two widespread nematodes across their geographic range. These differences are probably related to the differences in parasite life cycles, in which the development of parasite is differently dependent on the climate, especially important in the free-living infective stages. Therefore, the impact of these parasites on wild mustelid populations is likely to be mediated by a changing climate, with a potentially reduced prevalence and infection intensity, especially for *A. putorii*. This large-scale analysis of the variation in infection parameters provides an opportunity to better understand the factors limiting the infection and the impact of nematodes on host communities in relation to geographic location and climate change.

## Declaration of competing interest

The authors declare that they have no known competing financial interests or personal relationships that could have appeared to influence the work reported in this paper.
