# Supplementary material for: A tale of two nematodes: Climate mediates mustelid infection by nematodes across the geographical range
Source: Int J Parasitol Parasites Wildl. 2022 Feb 9;17:218–24. doi: 10.1016/j.ijppaw.2022.02.005 (PMC8883794; doi:10.1016/j.ijppaw.2022.02.005)

**Supplementary materials**

A tale of two nematodes: climate mediates mustelid infection by nematodes across the geographical range

Andrzej Zalewski^a,^*, Marta Kołodziej-Sobocińska^a^ and Kamil A. Bartoń^b^

**Table S1.** Selected literature data on *Aonchotheca putorii* (= syn. *Capillaria putorii*) and *Molineus patens* infection parameters: prevalence and mean infection intensity of the seven mustelid species considered in this study. Only studies with more than 5 samples were taken into account. ST– stomachs included in the analysis; NS – not specified parts of the alimentary tract analysed; NA – not analyse/ not available.

| Number  of hosts | Alimentary tract parts | ***Aonchotheca putorii*** | | | ***Molineus patens*** | | | References |
| --- | --- | --- | --- | --- | --- | --- | --- | --- |
|  |  | Prevalence  (%) | Mean infection  intensity | Prevalence  (%) | | Mean infection  intensity |  | |
| ***Martes americana*** | | |  |  | |  |  | |
| 42 | ST | 86.0 | 56.0 | NA | | NA | Foreyt and Lagerquist, 1993 | |
| 13 | ST | 54.0 | 9.8 | NA | | NA | Butterworth and Beverley-Burton, 1981 | |
| 140 | ST | 47.0 | NA | NA | | NA | Veine-Smith et al., 2011 | |
| 78 | NS | NA | NA | 9.0 | | 1.8 | Hoberg et al., 1990 | |
| 141 | ST | NA | NA | 7.8 | | 5.6 | Scranton, 1986 | |
| ***Martes foina*** | | |  |  | |  |  | |
| 67 | ST | 31.3 | NA | 1.5 | | NA | Pfeiffer et al., 1989 | |
| 103 | NS | 8.9 | NA | 32.7 | | NA | Miquel et al., 1993 | |
| 9 | ST | 42.9 | 20.3 | 14.3 | | 1 | Di Cerbo et al., 2005 | |
| 259 | ST | 86.9 | NA | 10.4 | | NA | Schoo et al., 1994 | |
| 7 | ST | 14.3 | 1.0 | NA | | NA | Varodi et al., 2017 | |
| 89 | NS | 13.8 | 2.0 | 37.5 | | 14.3 | Feliu et al., 1996 | |
| 10 | NS | 10.0 | 1.0 | 50.0 | | 1.1 | Millán and Ferroglio, 2001 | |
| 30 | NS | 20.0 | 8.2 | 13.3 | | 9.7 | Itin, 2014 | |
| ***Mustela lutreola*** | | |  |  | |  |  | |
| 45 | NS | 15.0 | 3.0 | 84.6 | | 15.2 | Torres et al., 2008 | |
| 28 | NS | 7.1 | 10.0 | 53.6 | | 9.8 | Torres et al., 2003 | |
| 17 | NS | 35.3 | 9.0 | 18.0 | | 10.0 | Sidorovich and Anisimova, 1999 | |
| 56 | NS | 12.5 | NA | 5.3 | | NA | Anisimova, 2004 | |
| 25 | NS | 8.0 | NA | NA | | NA | Abalikhin et al., 2019 | |
| 13 | ST | 30.8 | 4.3 | 69.0 | | 37.4 | Liberge, 2004 | |
| 30 | NS | 30.0 | 5.0 | 20.0 | | 9 | Shymalau et al., 1991 | |
| ***Martes martes*** | | |  |  | |  |  | |
| 102 | ST | 21.6 | 11.4 | 9.8 | | 7.7 | Segovia et al., 2007 | |
| 20 | NS | 30.8 | 5.5 | NA | | NA | Zhigileva and Uslamina, 2016 | |
| 87 | ST | 59.8 | 13.9 | 17.2 | | 2.4 | Maslennikova, 2010 | |
| 7 | ST | 42.9 | NA | NA | | NA | Soltys, 1962 | |
| 18 | ST | 33.3 | 9.5 | NA | | NA | Ruhlyadev and Ruhlyadeva, 1959 | |
| 96 | NS | 1.3 | NA | NA | | NA | Abalikhin et al., 2018 | |
| 20 | NS | 25.0 | 5.8 | 5.0 | | 7.0 | Itin, 2014 | |
| ***Mustela putorius*** | | |  |  | |  |  | |
| 68 | NS | 50.0 | 10.0 | 44.1 | | 16.5 | Torres et al., 1996 | |
| 37 | NS | 21.2 | 6.4 | 63.6 | | 45.5 | Torres et al., 2008 | |
| 10 | NS | 20.0 | 3.0 | 20.0 | | 5.5 | Nugaraite et al., 2014 | |
| 8 | ST | 50.0 | 63.4 | NA | | NA | Nugaraite et al., 2019 | |
| 18 | ST | 16.6 | 19.3 | NA | | NA | Nugaraite et al., 2019 | |
| 118 | ST | 79.7 | 45.0 | 95.8 | | 63.2 | Kretschmar, 2016 | |
| 76 | NS | 34.3 | 8.0 | 2.9 | | 2.0 | Anisimova and Poloz, 2017 | |
| 48 | NS | 8.3 | NA | NA | | NA | Abalikhin et al., 2019 | |
| 15 | ST | 6.7 | 18.0 | NA | | NA | Prokopic, 1958 | |
| 305 | NS | 1.0 | NA | NA | | NA | Prokopic, 1965 | |
| 40 | NS | 35.0 | 7.0 | 17.5 | | 4.0 | Shymalau et al., 1991 | |
| 15 | ST | 33.3 | NA | NA | | NA | Malczewski, 1964 | |
| ***Martes zibellina*** | | |  |  | |  |  | |
| 1124 | ST | 10.1 | 14.0 | 2.0 | | 3.0 | Gubanov, 1964 | |
| 100 | NS | 25.6 | 4.3 | NA | | NA | Zhigileva and Uslamina, 2016 | |
| 41 | NS | 32.0 | 4.7 | NA | | NA | Uslamina and Zhigileva, 2015 | |
| 462 | ST | 1.7 | NA | 3.46 | | NA | Kokolova and Illarionov, 2017 | |
| 305 | ST | 62.6 | 17.3 | 3.9 | | NA | Romanov, 1959, 1960 | |
| 94 | ST | 50.0 | NA | 19.1 | | NA | Sulimov, 1968 | |
| 510 | ST | 25.9 | 3.0 | 30.0 | | 10.0 | Kontrimavichus, 1969 | |
| 347 | NS | 3.2 | 3.6 | 0.3 | | 2.0 | Odnokurtsev and Sedalischev, 2011 | |
| 363 | NS | 6.9 | 5.4 | 2.7 | | 2.4 | Odnokurtsev and Sedalischev, 2011 | |
| 217 | NS | 8.3 | 5.3 | 0.5 | | 5.0 | Odnokurtsev and Sedalischev, 2011 | |
| 138 | NS | 5.1 | 4.7 | 1.4 | | 1.5 | Odnokurtsev and Sedalischev, 2011 | |
| 254 | NS | 2.4 | 9.0 | 1.2 | | 4.0 | Odnokurtsev and Sedalischev, 2011 | |
| 57 | NS | 1.7 | 6.0 | NA | | NA | Odnokurtsev and Sedalischev, 2011 | |
| 172 | NS | 2.9 | 4.6 | NA | | NA | Odnokurtsev and Sedalischev, 2011 | |
| ***Neovison vison*** | | |  |  | |  |  | |
| 50 | ST | 34.0 | 5.0 | 2.0 | | 1.0 | Zabiega, 1996 | |
| 45 | ST | 60.0 | 42.0 | NA | | NA | Butterworth and Beverley-Burton, 1981 | |
| 47 | ST | NA | NA | 2.1 | | NA | Dorney and Lauerman, 1969 | |
| 120 | NS | 20.0 | NA | 28.3 | | NA | Miller and Harkema, 1964 | |
| 48 | NS | 17.0 | 4.0 | NA | | NA | Jennings et al., 1982 | |
| 6 | ST | 33.3 | NA | NA | | NA | Romanov, 1960 | |
| 6 | ST | NA | NA | 16.7 | | 2.0 | Sulimov, 1968 | |
| 36 | ST | 58.3 | 10.0 | 33.3 | | 10.0 | Ivonin and Ivonina, 2019 | |
| 281 | ST | 1.4 | 1.0 | 50.1 | | 14.5 | Kontrimavichus, 1963 | |
| 56 | ST | 82.0 | 170.0 | 66.0 | | 32.0 | Kontrimavichus, 1969 | |
| 50 | NS | 20.0 | NA | 8.0 | | NA | Shimalov and Shimalov, 2001 | |
| 50 | NS | 12.0 | 5.3 | 36.0 | | 6.7 | Torres et al., 2003 | |
| 42 | NS | 18.4 | 9.4 | 50.0 | | 15.9 | Torres et al., 2008 | |
| 9 | NS | 33.0 | 9.1 | 33.0 | | 1.8 | Nugaraite et al., 2014 | |
| 50 | ST | 54.0 | 5.0 | 68.0 | | 8.0 | Martínez-Rondán et al., 2017 | |
| 20 | ST | 50.0 | 44.2 | 20.0 | | 10.0 | Nugaraite et al., 2014 | |
| 39 | ST | 33.3 | 31.8 | 12.8 | | 5.5 | Nugaraite et al., 2014 | |
| 69 | NS | 31.7 | 5.6 | 26.0 | | 6.7 | Itin and Krabchenko, 2017 | |
| 107 | NS | 2.8 | NA | NA | | NA | Abalikhin et al., 2019 | |
| 120 | NS | 5.0 | NA | NA | | NA | Konovalov et al., 2013 | |
| 20 | NS | NA | NA | 57.1 | | NA | Miquel et al., 1993 | |
| 13 | ST | 23.1 | 52.0 | NA | | NA | Varodi et al., 2017 | |
| 11 | NS | NA | NA | 45.4 | | 6.6 | Feliu et al., 1996 | |
| 12 | ST | 50.0 | 10.7 | 66.7 | | 12.9 | Liberge, 2004 | |
| 66 | ST | NA | NA | 15.2 | | 1.1 | Zschille et al., 2004 | |
| 39 | ST | 71.8 | 102.0 | NA | | NA | Maslennikova and Stelnikov, 2018 | |
| 122 | NS | 11.5 | 7.0 | 6.6 | | 2.0 | Shymalau et al., 1991 | |
| 133 | NS | 36.3 | 18.0 | 3.8 | | 6.0 | Anisimova and Poloz, 2010 | |
| 62 | NS | 25.8 | 8.5 | 9.7 | | 7.2 | Torres et al., 2003 | |

**References**

Abalikhin, B.G., Kryuchkova, E.N., Egorov, S.B., Sokolov, E.A., 2018. Helminths and diet composition of martens in Central region of RF. Agrarnyj Vestnik Verhnevolzhya 24, 103-106.

Abalikhin, B.G., Kryuchkova, E.N., Sokolov, E.A., 2019. The current situation analysis at the parasite fauna of semi-aquatic carnivores of the weasel family in the central region of the Russian Federation. Theory and Practice of Parasitic Disease Control: Collection of Scientific Articles adapted from the International Scientific Conference 20, 31-35.

Anisimova, E., 2004. Study on the European mink *Mustela lutreola* helminthocenoses in connection with the American mink *M. vison* expansion in Belarus: story of the study and review of the results. Helminthologia 41, 193-196.

Anisimova, E., Poloz, S., 2010. Parasitoses of American mink in wild populations and farming. Belaruskaya Navuka, Minsk.

Butterworth, E.W., Beverley-Burton, M., 1981. Observations on the prevalence and intensity of *Capillaria* spp. (Nematoda, Trichuroidea) in wild carnivora from Ontario, Canada. P. Helm. Soc. Wash. 48, 24-37.

Di Cerbo, A.R., Manfredi, M.T., Bregoli, M., Ferro Milone, N., 2005. Helminth fauna of mustelids in north-eastern Italy. Hystrix, It. J. Mamm. Supp. 16, 112.

Dorney, R.S., Lauerman, L.H., 1969. A helminthological survey of wild mink in Wisconsin. Bull. Wildl. Dis. Ass. 5, 35-36.

Feliu, C., Miquel, J., Casanova, J.C., Torres, J., Segovia, J.M., Fons, R., Ruiz Olmo, J., 1996. Helminthfaunas of wild carnivores in the Montseny massif: an atypical ecosystem in the Northeast of the Iberian peninsula. Vie et Milieu 46, 327-332.

Foreyt, W.J., Lagerquist, J.E., 1993. Internal parasites from the marten (*Martes americana*) in eastern Washington. J. Helminthol. Soc. W. 60, 72-75.

Gubanov, N.M., 1964. Helminth fauna of farm mammals of Yakutia. Nauka, 115.

Hoberg, E.P., Aubry, K.B., Brittell, J.D., 1990. Helminth parasitism in martens (*Martes americana*) and ermines (*Mustela erminea*) from Washington, with comments on the distribution of *Trichinella spiralis*. J. Wildl. Dis. 26, 447-452.

Itin, G., 2014. Features of helminthocenoses of wild carnivorous mammals in the landscape and geographical zones of the Northwest Caucasus. Krasnodar State University, Krasnodar.

Itin, G., Krabchenko, V.M., 2017. Ecological and faunistic characteristics of helminth communities in American mink (*Mustela vison*) in biocenoses of the North-West Caucasus. Theory and Practice of Parasitic Animal Diseases (Теория и практика паразитарных болезней животных) 18, 188-190.

Ivonin, Y.V., Ivonina, O.Y., 2019. Characteristics of the helmint fauna of the gastro - intestinal tract of the American mink (*Mustela vison* Schreber, 1777), in the basin of the river of Goloustnoye Irkutsk Region. Vestnik IrGSHA 93, 54-61.

Jennings, D.H., Threlfall, W., Dodds, D.G., 1982. Metazoan parasites and food of short-tailed weasels and mink in Newfoundland, Canada. Can. J. Zool. 60, 180-183.

Kokolova, L.M., Illarionov, A.I., 2017. Helminth fauna in sable (*Martes zibellina* Linnaeus, 1758) from Yakutia. Russ. J. Parasitol. 42, 330-333.

Konovalov, A.P., Sapozhnikova, A.I., Akbaev, M.S., 2013. Parasitofauna of furry animals and other carnivorous animals in the Central Districts of Non-Chernozem Zone and Volga-Vyatka Region. Russ. Vet. J. 9, 25-27.

Kontrimavichus, V.L., 1963. Helminths of American mink introduced in Habarovsk region. Izdatelstvo Akademii Nauk Kazahstana, Alma-Ata.

Kontrimavichus, V.L., 1969. Helminth fauna of Mustelids from Altai Mountains. Parasitologya 3, 406-410.

Kretschmar, F.M., 2016. Die Parasiten des Europäischen Iltisses *Mustela putorius* Linnaeus, 1758 in Deutschland. Ludwig-Maximilians-Universität, München, pp. 1-194.

Liberge, M., 2004. Statut parasitaire du vison d'Europe (*Mustela lutreola*) en France: Etude comparative de la faune helminthique du vison d'Europe, du vison d'Amérique (*Mustela vison*) et du putois (*Mustela putorius*).

Malczewski, A., 1964. A contribution to the knowledge of Mustelidae helminthofauna in Poland. Wiad. Parazytol. 10, 565-567.

Martínez-Rondán, F.J., Ruiz de Ybáñez, M.R., Tizzani, P., López-Beceiro, A.M., Fidalgo, L.E., Martínez-Carrasco, C., 2017. The American mink (*Neovison vison*) is a competent host for native European parasites. Vet. Parasitol. 247, 93-99.

Maslennikova, O.V., 2010. Helminth fauna of forest marten (*Martes martes* L.) in the Kirov area. Russ. J. Parasitol. 35, 29-40.

Maslennikova, O.V., Stelnikov, D.P., 2018. Parasitocenosis of the American mink at the urbanized ecosystems of Kirov region. Vestnik Permskogo Universiteta, Biologija 2, 182-187.

Millán, J., Ferroglio, E., 2001. Helminth parasites in stone martens (*Martes foina*) from Italy. Z. Jagdwiss. 47, 229-231.

Miller, G.C., Harkema, R., 1964. Studies on helminths of North Carolina vertebrates .V. Parasites of mink *Mustela vison* Schreber. J. Parasitol. 50, 717-720.

Miquel, J., Feliu, C., Torres, J., Casanova, J.C., 1993. Corología de las especies de nematodos parásitas de carnívoros silvestres en Cataluña (NE península ibérica). Miscellània Zoològica 17, 49-57.

Nugaraite, D., Mazeika, V., Paulauskas, A., 2014. Helminths of mustelids (Mustelidae) in Lithuania. Biologija 60, 117-125.

Nugaraite, D., Mazeika, V., Paulauskas, A., 2019. Helminths of mustelids with overlapping ecological niches: Eurasian otter *Lutra lutra* (Linnaeus, 1758), American mink *Neovison vison* Schreber, 1777, and European polecat *Mustela putorius* Linnaeus, 1758. Helminthologia 56, 66-74.

Odnokurtsev, V.A., Sedalischev, V.T., 2011. Helminthes fauna of sable (*Martes zibellina*, Linnaeus) in Yakutia. Tomsk State University Journal of Biology, 22-34.

Pfeiffer, A.S., Bockeler, W., Lucius, R., 1989. Parasites of the domestic and wild anomals of Schleswig-Holstein - parasites of the inner organs of the beech marten (*Martes foina*). Z. Jagdwiss. 35, 100-112.

Prokopic, J., 1958. Studium helmintofauny šelem v Čechách a na Moravě. Československa Parasitologie 5, 157-164.

Prokopic, J., 1965. The helminthofauna of Czechoslovak carnivores. Československa Parasitologie 12, 207-226.

Romanov, I.V., 1959. Dependence of sable helminthfauna in Krasnoyarsk Territory on the external medium of this animal. Zoologicheskii Zhurnal 38, 1313-1321.

Romanov, I.V., 1960. The helminth fauna of sables and other Mustelidae in the Krasnoyarsk Territory. Zoologicheskii Zhurnal 39 995-1002.

Ruhlyadev, D.P., Ruhlyadeva, M.N., 1959. Helminth parasites in martens and other predators of north-western Causasus. Trudy Kavkazskogo Gosudarstvennogo Zapovednika 5, 127-134.

Schoo, G., Pohlmeyer, K., Stoye, M., 1994. Zur Helminthenfauna des Steinmarders (*Martes foina* Erxleben 1777). Z. Jagdwiss. 40, 84-90.

Scranton, C.R., 1986. Parasites of pine marten, *Martes americana* in northeastern Alaska. Montana State University, Bozeman.

Segovia, J.M., Torres, J., Miquel, J., Sospedra, E., Guerrero, R., Feliu, C., 2007. Analysis of helminth communities of the pine marten, *Martes martes*, in Spain: Mainland and insular data. Acta Parasitol. 52, 156-164.

Shimalov, V.V., Shimalov, V.T., 2001. Helminth fauna of the American mink (*Mustela vison* Schreber, 1777) in Belorussian Polesie. Parasitol. Res. 87, 886-887.

Shymalau, U., Sidorovich, V.E., Shymalau, V.U., 1991. Helminths of riparian mustelids in Belarus. Vetsi Akademii Navuk Belarusi, Seryya Biyalagichnykh Navuk 4, 96-101.

Sidorovich, V., Anisimova, E.I.E., 1999. Comparative analysis of the helminthocenoses of the native semiaquatic Mustelids (*Lutra lutra*, *Mustela lutreola*) in Connection with the width of Food Spectra IUCN Otter Spec. Group Bull 16, 76-78.

Soltys, A., 1962. Helminth parasites of Mustelidae of the Lublin Palatinate Acta Parasitol. Polonica 10, 73-76.

Sulimov, A.D., 1968. Helminths of martens in Tuva. Zoologicheskii Zhurnal 47, 1089-1091.

Torres, J., Feliu, C., Miquel, J., Casanova, J.C., García-Perea, R., Gisbert, J., 1996. Helmintofauna de *Mustela putorius* Linnaeus, 1758 (Carnivora: Mustelidae) en la península Ibérica. Bolletí de la Societat d'Història Natural de les Balears 39, 155-165.

Torres, J., Manas, S., Palazon, S., Cena, J.C., Miquel, J., Feliu, C., 2003. Helminth parasites of *Mustela lutreola* (Linnaeus, 1761) and *M. vison* Schreber, 1777 in Spain. Acta Parasitol. 48, 55-59.

Torres, J., Miquel, J., Fournier, P., Fournier-Chambrillon, C., Liberge, M., Fons, R., Feliu, C., 2008. Helminth communities of the autochthonous mustelids *Mustela lutreola* and *M. putorius* and the introduced *Mustela vison* in south-western France. J. Helminthol. 82, 349-355.

Uslamina, I.M., Zhigileva, O.N., 2015. Morphology and ecological features of the sable *Martes zibellina*, inhabiting in the territory of the Tyumen Region, Fundamental and applied aspects of modern biology: materials of the 2nd Russian Youth Scientific Conference (Фундаментальные и прикладные аспекты современной биологии: материалы II Всероссийской молодежной научной конференции), Tomsk, Russia, p. 120.

Varodi, E.I., Malega, A.M., Kuzmin, Y.I., Kornyushin, V.V., 2017. Helminths of wild predatory mammals of Ukraine. Nematodes. Vestnik Zoologii 51 187-202.

Veine-Smith, A.M., Bird, J., Belant, J.L., 2011. Patterns of endoparasite infections in american martens (*Martes americana*) of the Upper Peninsula of Michigan, USA. Comp. Parasitol. 78, 225-233.

Zabiega, M.H., 1996. Helminths of mink, *Mustela vison*, and muskrats, *Ondatra zibethicus*, in southern Illinois. J. Helminthol. Soc. W. 63, 246-250.

Zhigileva, O.N., Uslamina, I.M., 2016. Helminths infestation of various mitochondrial lines of the sable *Martes zibellina* and the pine marten *M. martes*. Ekologicheskaya Genetika 14, 43-49.

Zschille, J., Heidecke, D., Stubbe, M., 2004. Verbreitung und Ökologie des Minks - *Mustela vison* Schreber, 1777 (Carnivora, Mustelidae) - in Sachsen-Anhalt. Hercynia 37, 103-126.

**Table S2**. Selection tables for models of prevalence and infection intensity. All models included interaction of parasite species with the explanatory variables specified in the first column. All-subsets of explanatory variables were included, with a restriction to a maximum of 5 variables in a model. In addition, combinations of intercorrelated variables, with (G)VIF ≥ 5, were excluded. The table shows model spcecification, ΔAIC_c_ and the model weight derived from it. Models were ranked with AIC_c_. Only models with ΔAIC_c_ ≤ 4 are shown. ‘k’ – number of model parameters, ‘ΔAIC_c_’ – relative AIC_c_ (difference of model’s AIC_c_ to the lowest one), ‘weight’ – model weight (normalized to sum to 1 over the whole set of models). Part – the analysed parts of the alimentary tract: stomach or part were not specified; TempYear – average temperature over the whole year, SeasonalPrec and SeasonalTemp – seasonality of precipitation and temperature, respectively, PrecWarm, PrecCold, TempWarm and TempCold – precipitation and temperature in warmest and coldest quarters.

| **Model** | **k** | **ΔAIC*_c_*** | **weight** |
| --- | --- | --- | --- |
| *Prevalence* |  |  |  |
| Part + SeasonalPrec + TempCold + TempWarm | 11 | 0.1 | 0.3 |
| Part + SeasonalTemp + SeasonalPrec + TempWarm | 11 | 0.2 | 0.3 |
| Part + SeasonalPrec + TempYear + TempWarm | 11 | 0.7 | 0.2 |
| Part + SeasonalTemp + SeasonalPrec | 9 | 3.4 | 0.1 |
| Part + SeasonalPrec + TempCold | 9 | 3.5 | 0.1 |
| *Infection intensity* |  |  |  |
| Part + SeasonalPrec + TempYear + TempWarm | 10 | 0.0 | 0.3 |
| Part + SeasonalTemp + SeasonalPrec + TempWarm | 10 | 0.3 | 0.2 |
| Part + SeasonalPrec + TempCold + TempWarm | 10 | 0.4 | 0.2 |
| Part + SeasonalPrec + TempWarm | 8 | 2.5 | 0.1 |
| Part + PrecWarm + SeasonalPrec + TempWarm | 10 | 2.8 | 0.1 |
| Part + SeasonalTemp + PrecCold + TempWarm | 10 | 3.7 | 0.0 |
| Part + PrecCold + TempCold + TempWarm | 10 | 3.7 | 0.0 |
| Part + TempCold + TempWarm | 8 | 3.8 | 0.0 |
| Part + SeasonalTemp + TempWarm | 8 | 3.8 | 0.0 |
| Part + TempYear + TempWarm | 8 | 3.9 | 0.0 |

**Fig. S1.** Correlation between the two parasite infection measures analysed in this study: mean infection intensity and prevalence of the two nematode species. Lines denote a Reduced Major Axis fit (mean and 95% confidence intervals). Note the log-scale on y-axes. Pearson's correlation coefficients: for *Aonchotheca putorii* r_p_ = 0.67 (p ≪ 0.001), for *Molineus patens* r_p_ = 0.22 (p = 0.2).


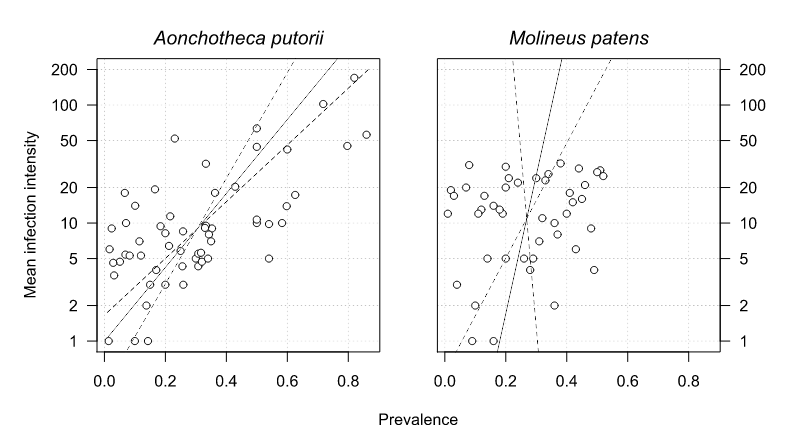

Supplement: Multimedia component 1 [file mmc1.docx]
